# Diffuse glioma perfusion quantification with ASL and DSC: a head-to-head comparison with ^15^O-H_2_O PET

**DOI:** 10.3389/fonc.2026.1766519

**Published:** 2026-08-05

**Authors:** Jan Petr, Niels Verburg, Joost P. A. Kuijer, Thomas Koopman, Vera C. Keil, Esther A. H. Warnert, Frederik Barkhof, Joerg van den Hoff, Ronald Boellaard, Philip C. De Witt Hamer, Henk J. M. M. Mutsaerts

**Affiliations:** 1 Helmholtz-Zentrum Dresden-Rossendorf, Institute of Radiopharmaceutical Cancer Research, Dresden, Germany; 2 Department of Radiology and Nuclear Medicine, Amsterdam University Medical Center, Vrije Universiteit Amsterdam, Amsterdam, Netherlands; 3 Department of Neurosurgery, Amsterdam University Medical Center, Amsterdam, Netherlands; 4 Brain Tumor Center Amsterdam, Cancer Center Amsterdam, Amsterdam University Medical Center, Amsterdam, Netherlands; 5 Department of Radiology and Nuclear Medicine, Erasmus Medical Center, Rotterdam, Netherlands; 6 Queen Square Institute of Neurology and Centre for Medical Image Computing, University College London, London, United Kingdom

**Keywords:** ASL, DSC, glioma, perfusion, PET

## Abstract

**Background and purpose:**

Arterial spin labeling (ASL) is a non-invasive alternative to dynamic susceptibility contrast (DSC) for measuring cerebral blood flow (CBF), even though both methods measure slightly different contrast. This study compares ASL and DSC MRI with the gold-standard measurement ^15^O-H_2_O PET in gliomas.

**Methods:**

Eight patients (age 40.5 ± 17.0 years, 3 women) with grade 2–4 gliomas were scanned at 3T MRI with ASL and DSC, and at PET using a ^15^O-H_2_O PET tracer. Quantitative comparison was performed in contralateral normal-appearing tissue, and in tumors with and without normalization to contralateral tissue. The mean voxel-wise relative error (MRE) was calculated for PET-ASL and PET-DSC. The MRE difference between ASL and DSC was evaluated using a t-test (p < 0.05 after Bonferroni correction).

**Results:**

ASL showed superior (p<0.001) voxel-wise agreement with PET (MRE 26.8%) in the normal-appearing tissue compared with DSC (MRE 33.8%). While the tumor maximum CBF normalized to contralateral gray matter comparison with PET did not differ between (p=0.5) ASL (MRE 23.2%) and DSC (MRE 22.0%), voxel-wise agreement was better (p<0.001) for ASL (MRE 27.2%) than for DSC (MRE 35.0%). Visually, ASL overestimated CBF near larger arteries, and DSC underestimated CBF in non-enhancing tumors with tiny capillaries.

**Conclusion:**

While neither ASL nor DSC is fully comparable to ^15^O-H_2_O PET in all tumors, ASL presents a viable non-invasive alternative to DSC for glioma imaging. These findings encourage future studies to investigate the extent to which the combination of ASL and DSC can measure complementary tumor hemodynamics.

## Introduction

While histopathology is the gold standard for assessing the malignancy of a brain tumor based on the extent of angiogenesis (1), MRI is a much more practical modality for repeated evaluation on a fine spatial resolution (2). One commonly used biomarker of tumor malignancy is perfusion MRI. Cerebral perfusion — defined as the volume of blood per unit time passing through a volume of brain tissue — is potentially a proxy biomarker for angiogenesis and the metabolic demand of proliferating tumor cells. Perfusion MRI can be used clinically in gliomas for tumor grading (3, 4), treatment evaluation (5, 6), or monitoring adverse effects of treatment (7–9), and tumor infiltration imaging with perfusion MRI was also shown in animal models (10).

While ^15^O-H_2_O PET is considered the gold standard for cerebral blood flow (CBF) imaging, it is not clinically used due to its short tracer half-life and limited availability. A more practical alternative is T2*-weighted dynamic MRI during the passage of a gadolinium-based contrast agent, referred to as dynamic susceptibility contrast (DSC) imaging. DSC estimates CBF by measuring intravascular mean transit time and relative blood volume. As DSC is sensitive to extravascular signals, its reliability for CBF quantification is affected by vascular changes such as blood-brain-barrier disruption (11). Moreover, standard DSC models provide relative values only, and normalization to a reference region is needed (11). Arterial spin labeling (ASL) MRI is a non-invasive alternative that uses magnetically labeled blood as an endogenous tracer. Similar to ^15^O-H_2_O PET, ASL directly measures CBF and may provide complementary information to DSC (12).

The accuracy and reproducibility of ASL CBF have been shown to be close to that of ^15^O-H_2_O PET in healthy young and older persons, in Moyamoya disease, stroke, and Alzheimer’s disease (13). Although comparable CBF was shown for both techniques for young and older adults, these perfusion modalities seem to differ in regions with delayed blood arrival (14). This delayed blood arrival is not only related to normal aging but can potentially occur as an effect of tumor neovascularization. While both DSC and ASL can be applied clinically (4, 15), they may provide distinct and complementary information in the presence of tumor vascular changes.

To determine the accuracy of ASL as a noninvasive alternative to DSC for glioma imaging, we compared CBF images of ASL and DSC with those of ^15^O-H_2_O PET in participants diagnosed with diffuse glioma. Firstly, we quantitatively compared the three modalities in normal-appearing tissue to validate that the accuracy in this tissue is similar to that in healthy tissue reported in previous studies. Secondly, we quantitatively compared the modalities within the tumor at the region- and voxel-wise levels. Finally, we qualitatively compared the modalities in the tumor region to identify potential pathophysiological explanations for any observed differences in accuracy.

## Methods

### Study population

Data were drawn from the *FRONTiers in advanced Imaging of unExplored glioma Regions (FRONTIER)* study. Glioma patients scanned within 14 days before surgery were prospectively included between September 2014 and February 2016 (16). Patients who did not undergo a ^15^O-H_2_O PET scan (n=12) were excluded from the current analysis. Other than general MRI contraindications, criteria for exclusion were pregnancy, other brain pathology on MRI, or a history of brain surgery, cranial irradiation, or chemotherapy. The study protocol received approval from the Medical Ethics Review Committee of the Free University Medical Center and was registered in the Dutch National Trial Register (NTR5354) (16). All participants provided written informed consent.

### Image acquisition

**Figure 1:**
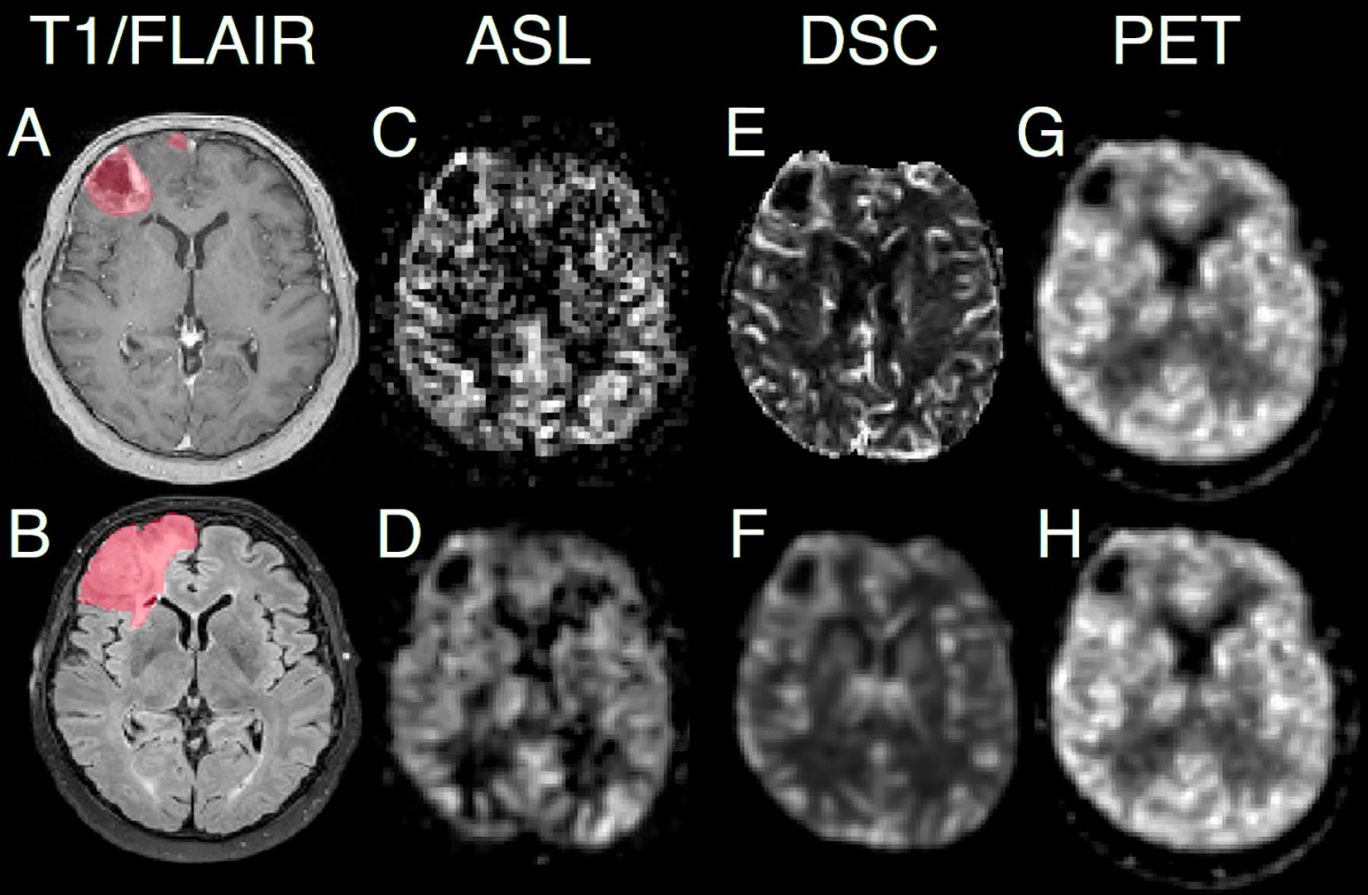
Single transverse slice for a representative case (participant 2, glioblastoma, IDH wild-type, male, 66y). **(A)** post-gadolinium T1-weighted image with overlaid delineated lesion and **(B)** T2-FLAIR image with overlaid segmented edema; **(C, D)** arterial spin labeling (ASL), **(E, F)** dynamic susceptibility contrast (DSC), **(G, H)** positron emission tomography (PET) images. The top row **(A– D)** shows the original images in native resolution for similar slice positions. The bottom row **(E–H)** shows the aligned images smoothed to the common 5.5x5.5x5.5 mm^3^ resolution. ASL, arterial spin labeling; DSC, dynamic susceptibility contrast; GBM, glioblastoma.

All MR imaging was performed using a 3T Achieva scanner (Philips Healthcare, Best, The Netherlands) equipped with an 8-channel Invivo head coil. Dynamic whole-brain PET scans were acquired on a Gemini time-of-flight PET-CT scanner (Philips Healthcare, Best, The Netherlands). The full acquisition protocol is detailed elsewhere (16). It included pseudo-continuous ASL (PCASL), DSC, ^15^O-H_2_O PET, T2-weighted FLAIR, and post-gadolinium 3D T1 (post-Gd T1w) (**Figure 1**). All imaging was performed prior to surgery, and none of the patients received neoadjuvant treatment.

PCASL was acquired by a 2D echo-planar imaging (EPI) readout, repetition time (TR)/echo time (TE) 4060 ms/14 ms, field of view (FOV) 240×240 mm^2^, matrix 80×80, 23 contiguous 5 mm slices, labeling duration 1650 ms, single post-labeling delay (PLD) 1525 ms, slice time 40 ms — leading to effective PLD range 1525–2405 ms from the first to the last slice — and two background suppression pulses. The labeling plane was positioned at the mid-level of the extracranial internal carotid arteries as identified on a 2D time-of-flight angiography. A separate M0 scan was acquired with TR 2000 ms — without labeling or background suppression — but otherwise with an identical readout.

DSC was acquired by a 2D gradient-echo EPI with TR/TE/flip angle 1600/40 ms/75° (participant 1), and 1900/30 ms/60° (participants 2-8), FOV 240×240 mm^2^, matrix 128×128, 31 contiguous 4 mm slices, 40 repetitions including 10 pre-contrast volumes. A pre-loading bolus of 0.05 mmol gadolinium contrast agent/kg body weight (Dotarem, Guerbet, 0.1 mL/kg body weight) was administered before DSC, followed by a bolus injection of 0.05 mmol/kg Dotarem during scanning, delivered at a rate of 5 mL/s.

A 3D T2-weighted FLAIR sequence with TR/TE/inversion time (TI) 4800/279/1650 ms, voxel size 1.12x1.12x1.12 mm^3^, and a 3D post-Gd T1w sequence with TR/TE/flip angle 7/3 ms/12°, and voxel size 0.98x0.98x1.0 mm^3^ were obtained.

PET scans were acquired dynamically for 10 min after intravenous administration of 370 MBq of ^15^O-H_2_O with simultaneous online arterial blood sampling at a rate of 5 mL/min with 3 manual 9 mL samples withdrawn at 5, 7, and 9 min after injection (**Figure 1D**).

### CBF quantification

PCASL images were processed with ExploreASL (17) and quantified according to the consensus recommendations (18). DSC images were analyzed using the Olea Sphere 3.0 software (Olea Medical, La Ciotat, France) with arterial input function manually selected in the contralateral middle cerebral artery and leakage correction used (19). PET quantification used kinetic modeling of the dynamic PET images and the arterial blood samples (20). The scans were acquired in list mode and reconstructed into 26 frames of 1 × 10 s, 8 × 5 s, 4 × 10 s, 2 × 15 s, 3 × 20 s, 2 × 30 s, 6 × 60 s. The row action maximum likelihood algorithm (RAMLA) was used for reconstruction of the scans with an isotropic voxel size of 2 mm. Thereafter, the dynamic images were smoothed using an isotropic 5 mm FWHM Gaussian kernel. CBF was calculated voxelwise using the basis function method. A single-compartment model was employed including an additional arterial blood volume parameter but without dispersion correction. The measured arterial tracer concentration was corrected for delay (20). Manual samples were used to calibrate the online detection system.

### Image processing

Tumor regions of interest (ROI) were manually delineated on post-Gd T1w, combining the central necrosis and the enhancing periphery into a single ROI (**Figure 1**). In non-enhancing tumors, all FLAIR abnormalities were delineated. T1-weighted images were segmented using CAT12 to obtain gray matter (GM) and white matter (WM) partial volume maps. The tumor ROI was excluded from the segmentation (21).

All ASL, DSC, and PET images were registered to the post-Gd T1w images using rigid-body registration and CBF/GM contrast (22). Subsequently, ASL and DSC images were resampled to the PET native space and smoothed to an effective spatial resolution 5.5x5.5x5.5 mm^3^, assuming Gaussian point spread functions (PSF) (23) (**Figure 1**).

To compare the effect of intensity normalization, four CBF maps were created for each modality: absolute CBF without normalization (CBF_abs_), and normalized CBF (CBF_GMnorm_, CBF_WMnorm_, CBF_ContrNorm_) to designate CBF maps normalized to the mean CBF of the following reference regions, respectively:

- *ROI_GM_* - GM in the hemisphere contralateral to the tumor — GM thresholded at 70%;
- *ROI_WM_* - deep WM in the hemisphere contralateral to the tumor — WM thresholded at 70% and eroded with one voxel (24);
- *ROI_Contr_* - an ROI that mirrored the tumor ROI to the contralateral hemisphere.

GM and WM map thresholding was performed in the 5.5x5.5x5.5 mm^3^ space.

### Quantitative normal-appearing tissue comparison

To validate that the accuracy in normal-appearing tissue is similar to healthy tissue from previous studies, CBF was compared in normal-appearing GM in the contralateral hemisphere (ROI_GM_). Mean-ROI CBF_abs_ was compared between ASL and PET, but not for DSC, as it provides relative values only. For this purpose, linear regression and within-subject coefficient of variation (wsCV) were used to quantify the general agreement and individual differences between modalities. Histogram comparisons within ROI_GM_, ROI_WM_, and ROI_GM_ were performed on the normalized CBF maps CBF_GMnorm_, CBF_WMnorm_, and CBF_abs_ (for ASL only), respectively, by calculating voxel-wise mean relative error (MRE).

### Quantitative tumor ROI comparison

The absolute (CBFabs) and normalized (CBFGMnorm, CBFWMnorm, CBFContrNorm) perfusion values were assessed in the tumor ROI to evaluate three commonly used methods for CBF normalization. To perform both clinically meaningful and detailed comparisons, we calculated the maximum value in the tumor and a voxel-wise error in the tumor ROI. ROI maximum (95th percentile was used to reduce the influence of noise) was compared for ASL and DSC with PET as the reference by using linear regression with zero intercept and wsCV. For a voxel-wise comparison, the MRE was calculated.

In ASL, vascular label collections — e.g., in dilated vessels — can lead to biased CBF quantification far exceeding values in normal macrovascular artifacts (ASL-CBF above 300 mL/min/100 g). To avoid biasing quantitative analyses, participants with very high ASL-CBF attributable to slower label arrival due to congestion in dilated draining veins were excluded from the calculation of linear regression, wsCV, and MRE.

### Qualitative tumor ROI comparison

After the quantitative analysis, we performed a whole-brain visual comparison between ASL, DSC, and PET to help interpret the quantitative findings.

### Statistical analysis

Linear regression was used to compare the mean and maximum regional values in normal tissue and tumor ROI, respectively, between ASL/DSC and PET. For the slope of the linear regression, a 95% confidence interval was calculated, and an adjusted R^2^ was calculated to compare the pair values between two modalities. A voxel-wise MRE was calculated for each subject, and a t-test was used to compare the error between ASL and DSC. Bonferroni correction for multiple comparisons was used to account for testing both absolute and normalized CBF, with significance set to p=0.05. Additionally, Lin’s concordance correlation coefficient was calculated to address issues with bias in the mean and variance.

## Results

Eight adult participants (5 men, age 40.5 ± 17.0 years) suspected of a supratentorial diffuse glioma with an indication for resective surgery, confirmed by the multidisciplinary neuro-oncology tumor board, were included in the study (**Table 1**).

**Table 1:** Detailed demographics and tumor grades for all participants.

Participant	Sex	Age	Location	Histopathology	Molecular status	WHO grade
1	F	28	left frontal	glioblastoma	IDH mutant	4
2	M	66	right frontal	glioblastoma	IDH wildtype	4
3	M	37	right frontal	astrocytoma	IDH mutant	2
4	F	38	left frontal	glioblastoma	IDH mutant	4
5	M	23	right parietal	oligodendroglioma	IDH mutant	2
6	M	20	left temporal	astrocytoma	IDH mutant	2
7	M	57	left parietal	glioblastoma	IDH wildtype	4
8	F	55	right occipital	glioblastoma	IDH wildtype	4

F/M, female/male; IDH, Isocitrate dehydrogenase; WHO, World Health Organization (by WHO 2016 classification criteria).

### Quantitative normal-appearing tissue comparison

**Figure 2:**
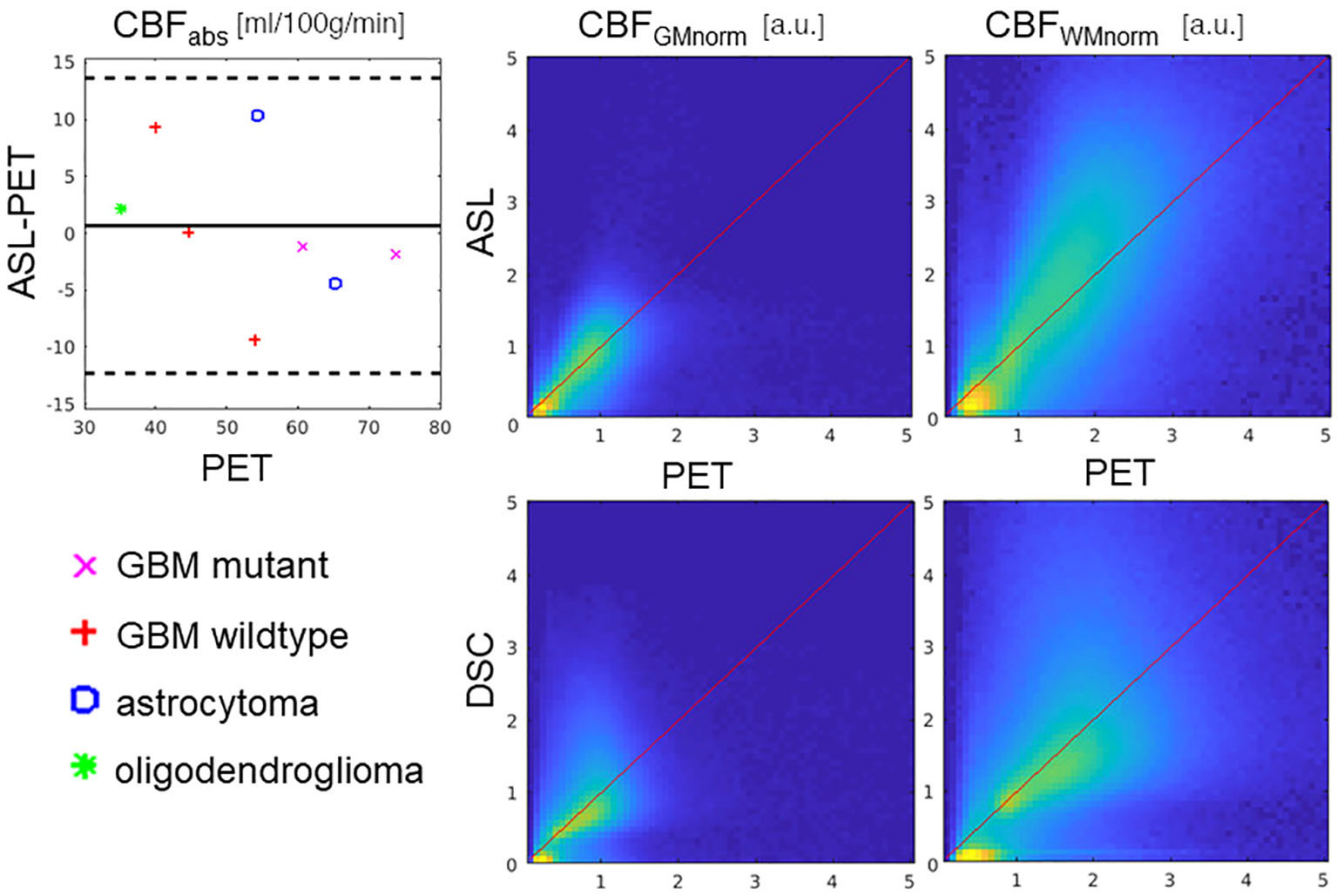
Comparison of mean CBF in the normal hemisphere for ASL and DSC with PET. A Bland-Altman plot of the mean of CBF_abs_ (in ml/100g/min) in GM is shown for individual participants on the left for ASL vs PET. A similar DSC vs PET comparison is not provided as DSC quantification provides only relative values. On the right, CBF_GMnorm_ and CBF_WMnorm_ (normalized, arbitrary units) difference between ASL and DSC with PET is shown as a joint histogram at the voxel level across all participants. ASL, arterial spin labeling; CBF, cerebral blood flow; DSC, dynamic susceptibility contrast; GBM, glioblastoma.

The linear regression for CBF_abs_ was: PET = 0.995*ASL (confidence interval (CI) 0.893-1.097, p<0.001, adjusted R^2^ 0.7), wsCV 12.4% (**Figure 2**), and Lin’s CC 0.879 (CI 0.868-0.889). Voxel-wise MRE in CBF_abs_, CBF_GMnorm_, and CBF_WMnorm_ between ASL and PET was 28.1%, 26.8%, and 34.6%, respectively; and 33.8% and 33.7% for CBF_GMnorm_ and CBF_WMnorm_, respectively, for DSC vs PET (both differences between ASL and DSC were significant with p<0.001 after Bonferroni correction).

### Quantitative tumor ROI comparison

**Figure 3:**
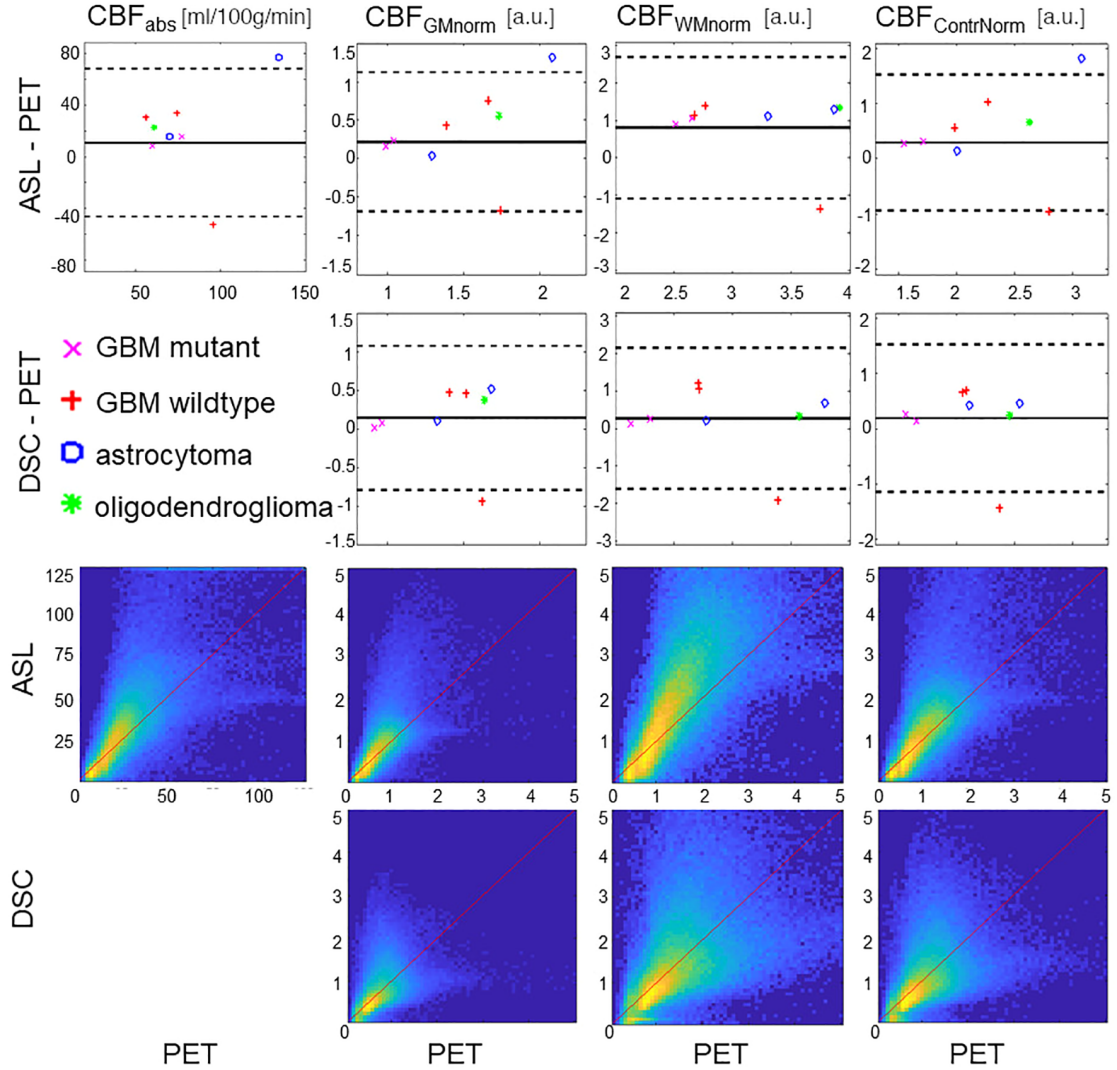
Comparison of ASL/DSC vs PET in tumor ROI. The upper two rows show the maximum of CBF for ASL vs PET (1st row) and DSC vs PET (2nd row). The bottom two rows show the joint histograms of CBF. The first column shows the absolute CBF_abs_ values (in ml/100g/min) for ASL only, and the legend lists the tumor types. The remaining columns show CBF values normalized (arbitrary units) to contralateral whole-hemisphere GM CBF (CBF_GMnorm_), contralateral WM CBF (CBF_WMnorm_), and mean CBF in the mirrored tumor ROI (CBF_ContrNorm_). Note that variability decreases with normalization for both methods, and this decrease is slightly greater with normalization to GM and the contralateral ROI than with normalization to contralateral WM. In terms of between-modality accuracy relative to PET, all three normalizations perform equally well for DSC. Normalization to WM appears to bias the ASL-PET comparison, although the histogram shape remains similar. ASL, arterial spin labeling; CBF, cerebral blood flow; DSC, dynamic susceptibility contrast; GBM; glioblastoma; GM, gray matter; ROI, region of interest; WM, white matter.

**Figure 4:**
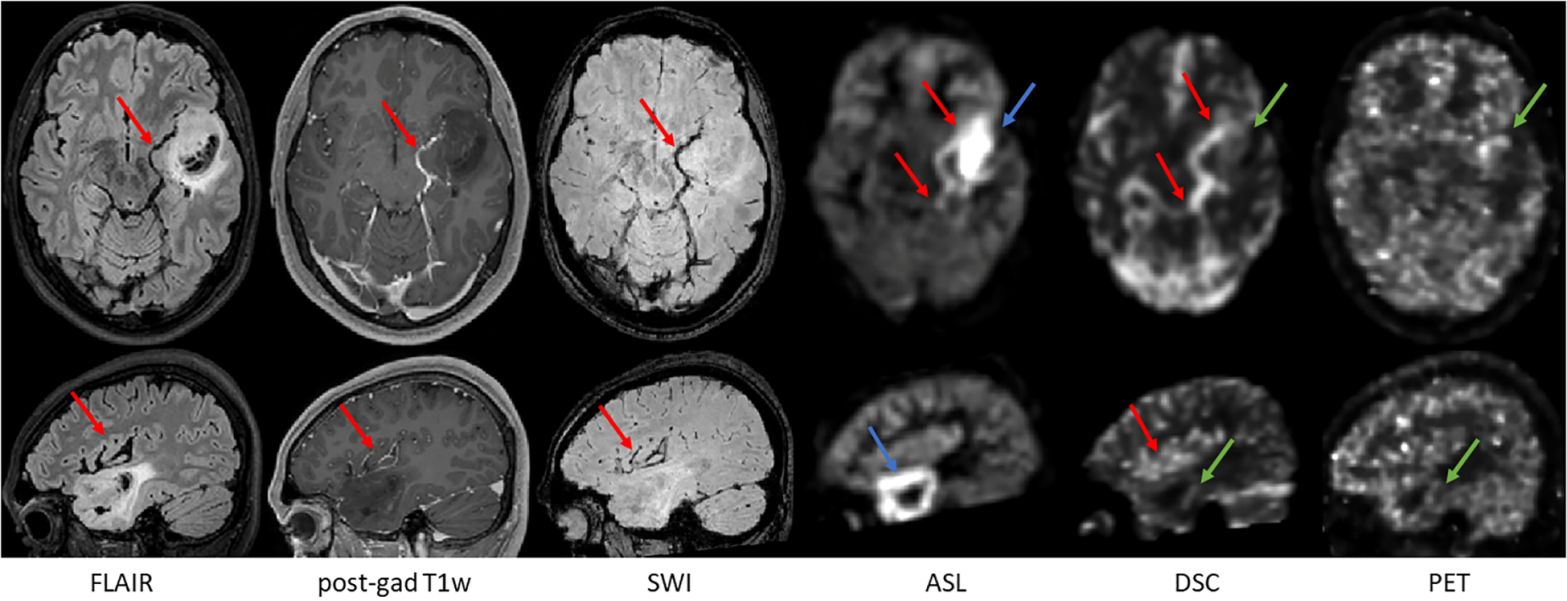
Participant 6 (grade 2 astrocytoma, IDH mutant, male, 20y) - a pair of representative axial (top row) and sagittal (bottom row) slices. On post-gadolinium T1w, the tumor shows inhomogeneous peripheral enhancement. On the axial images, a dilated draining vein is visible medial to the tumor on FLAIR, T1w, SWI, ASL, and DSC (red arrow). While the flow is likely high through this dilated vessel, the velocity is likely relatively low. This leads to a long arrival time, making the vessel visible on ASL and DSC (as indicated by the two red arrows), despite the absence of other macrovascular artifacts on ASL. On the sagittal images, a convolution of displaced and dilated M1 and M2 vessels running right above the tumor is visible on FLAIR, T1w, and SWI, with increased signal also on DSC, but not on ASL and PET (red arrow). Blood flow and volume comparable to that of normal GM were present in the tumor rim as assessed on PET and DSC (green arrows), and on the arterial blood volume (aBV) image of PET (not shown), respectively. ASL shows a very high signal in the entire tumor rim (over 300 mL/min/100g) (blue arrows). This CBF overestimation (normal GM CBF values are around 60mL/min/100g and lower than 100mL/min/100g) in ASL might be explained by the presence of numerous macroscopic vessels in the rim, in combination with delayed blood arrival. ASL, arterial spin labeling; CBF, cerebral blood flow; DSC, dynamic susceptibility contrast; GBM; glioblastoma; GM, gray matter; ROI, region of interest; WM, white matter.

Quantitative comparisons of maximum CBF within the tumor ROI are shown in **Table 2**; **Figure 3** shows the individual comparisons and joint histograms. Participant 6 was excluded from the quantitative analysis due to the presence of strongly dilated vessels next to the tumor (CBF 300–500 mL/min/100g) (**Figure 4**).

**Table 2:** Comparison between ASL and PET, and DSC and PET for absolute (CBF_abs_) and normalized CBF (CBF_GMnorm_, CBF_WMnorm_, CBF_ContrNorm_).

Method	Parameter	max CBF slope (CI)	max CBF p-value	max CBF wsCV	Voxel-wise CBF MRE	Lin’s CC CC (CI)
ASL	CBF_abs_	1.02 (0.6 -1.44)	0.001	41.5%	29.4%	0.45 (0.42-0.49)
ASL	CBF_GMnorm_	1.11 (0.76-1.45)	<0.001	32.9%	27.2%	0.53 (0.50-0.56)
ASL	CBF_WMnorm_	1.2 (0.82-1.58)	<0.001	31.3%	35.2%	0.28 (0.25-0.31)
ASL	CBF_ContrNorm_	1.08 (0.77-1.38)	<0.001	29.5%	28.2%	0.67 (0.65-0.69)
DSC	CBF_GMnorm_	1.05 (0.75-1.36)	<0.001	34.4%	35.0%	0.48 (0.44-0.52)
DSC	CBF_WMnorm_	1.03 (0.74-1.32)	<0.001	32.9%	37.0%	0.34 (0.29-0.37)
DSC	CBF_ContrNorm_	1.03 (0.74-1.32)	<0.001	32.7%	35.7%	0.15 (0.11-0.20)

The linear regression parameters and coefficient of variation (wsCV) are reported for all participants for ASL-DSC versus PET differences in maximum tumor CBF. Mean relative error (MRE) of CBF difference between ASL/DSC and PET is calculated across the population voxel-wise in the entire tumor ROI.

**Figure 5:**
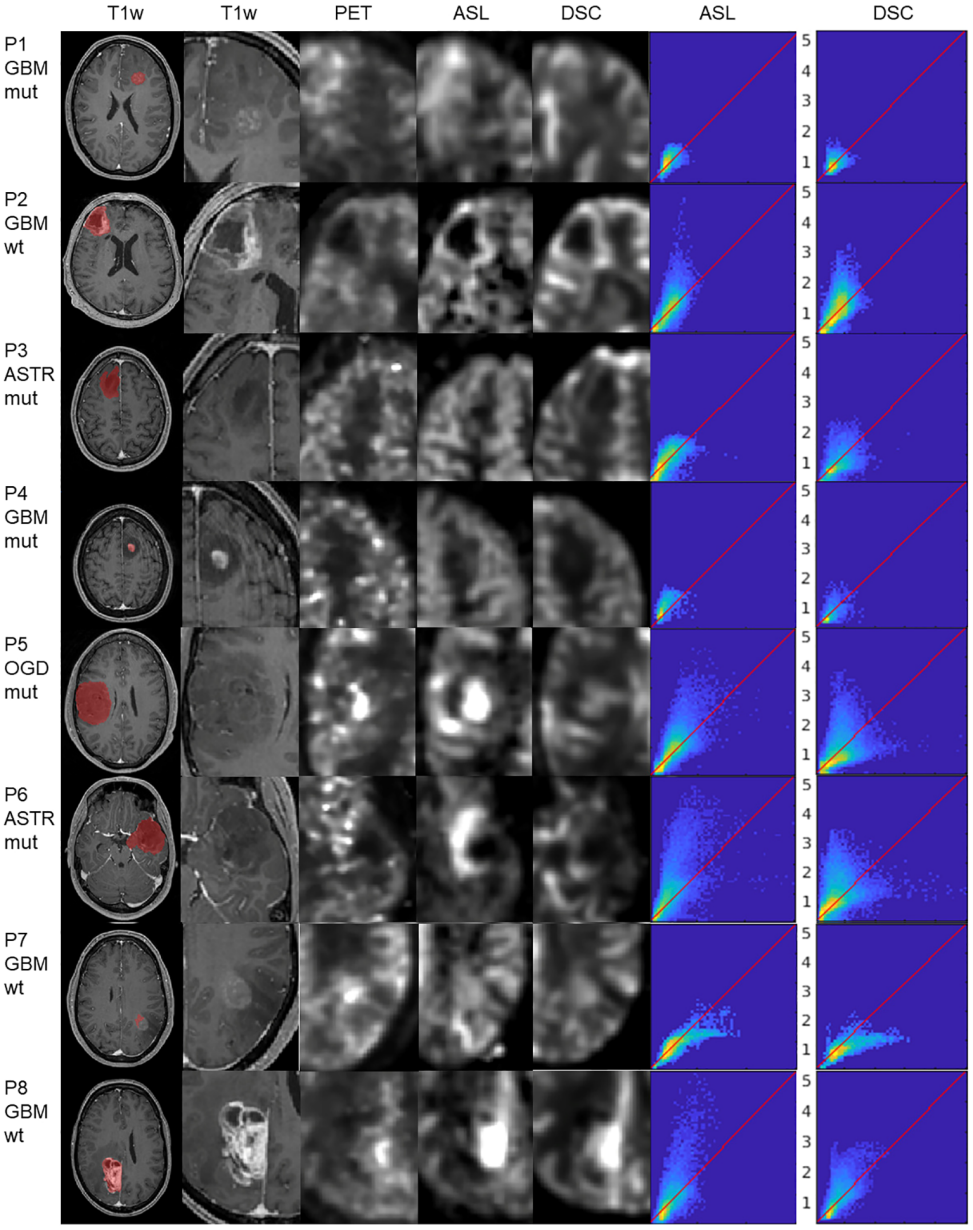
The figure shows a representative slice from each participant, including the tumor. The first column shows the T1-weighted whole-brain image in native resolution with the tumor ROI highlighted in red. The following columns show cropped regions including the tumor T1 for the following sequences: T1-weighted post gadolinium in native resolution; PET-CBF, ASL-CBF, and DSC-CBF, all smoothed to PET native resolution; and joint histograms of CBF_GMnorm_ over the tumor ROI comparing ASL-PET and DSC-PET (the scale of the histograms is 0–5 representing the normalized CBF values). ASL, arterial spin labeling; CBF, cerebral blood flow; DSC, dynamic susceptibility contrast; GBM; glioblastoma; GM, gray matter; ROI, region of interest; WM, white matter.

Both wsCV and Lin’s CC in maximum CBF in the tumor improved by normalization for ASL across all normalization methods except for normalization to WM. Voxel-wise analyses of normalized CBF showed reasonable agreement of both ASL and DSC with PET, though ASL tended to overestimate CBF for higher normalized CBF (>1.5), most commonly due to the presence of arterial transit artifacts (**Figure 5**), and DSC showed both regions of CBF overestimation and regions of no contrast accumulation despite being well-perfused according to PET (**Figure 5**). Voxel-wise MRE of CBF in comparison with PET was lower for ASL than with DSC (p<0.001 after Bonferroni correction). Lin’s CC showed poor concordance for both ASL and DSC, but ASL had a higher concordance than DSC for all types of normalization.

### Qualitative tumor ROI comparison

Participants 1, 3, 4, and 8 showed consistent CBF patterns across the three modalities (**Figure 5**). Participant 8 showed consistently increased perfusion in the solid right parafalcal tumor component, even though PET showed a smaller region with increased CBF. IDH-mutant participants 1, 3, and 4 showed lower tumor CBF than in the contralateral GM CBF. Participants 1 and 4 showed no hyperperfusion despite the presence of contrast-enhancing areas. The other four participants illustrate the substantial variability of findings for the three techniques. Participant 7 presented a sharp tumor hyperperfusion on PET, but only mild to no hyperperfusion on ASL or DSC. Conversely, participant 2, with an enhancing rim of the tumor, showed visible hyperperfusion on both ASL and DSC, but not on PET. Participant 6, with a histologically proven grade 2 astrocytoma, which had inhomogeneous peripheral contrast enhancement, was the only one with highly dilated vessels visible on susceptibility-weighted imaging (SWI) next to the tumor (**Figure 4**). DSC and PET showed tumor perfusion comparable to perfusion in normal-appearing GM, with the dilated draining vein clearly visible on DSC. On ASL, a rim around the whole tumor had very high values (200–400 mL/min/100g). Finally, participant 5, with a largely non-enhancing, non-necrotic-appearing tumor, revealed marked hyperperfusion on both PET and ASL but not clearly on DSC. Noteworthy in this participant is also the rim-like hyperperfusion in the periphery, indicating the frontlines of tumor expansion.

## Discussion

This study has three main findings. Firstly, ASL agreement with ^15^O-H_2_O-PET in normal-appearing tissue was comparable to previous volunteer studies and showed better voxel-wise agreement than DSC. Secondly, a similar agreement was observed in the tumor’s maximum normalized CBF between ASL and DSC, compared with ^15^O-H_2_O-PET. Contrary to DSC, intensity normalization to normal-appearing GM or to a contralateral ROI, rather than to normal-appearing WM, seems beneficial for ASL. Thirdly, ASL showed better qualitative agreement with PET inside tumor regions than DSC. In general, ASL tended to overestimate CBF in the presence of an intravascular signal, and DSC tended to underestimate perfusion in non-enhancing tumors.

The good agreement between ASL and PET in contralateral GM (slope of the linear regression of 1.00 (CI 0.89-1.10)) is similar to previous healthy-young-volunteer results (1.0 (CI 0.94–1.06)), with wsCV (12.4%) even being slightly better (17.3%) (25), suggesting that ASL CBF quantification accuracy is not compromised in glioma participants in normal-appearing tissue. Lin’s CC, which also considers the intercept, was poor for both methods, partly also due to the low number of subjects. However, it confirmed that GM or contralateral normalization is superior to WM normalization for ASL. And showed better agreement of ASL with PET than DSC. Voxel-wise results in normal tissue suggest that normalization to GM or WM improves the inter-modality difference for DSC, but only GM normalization is advantageous for ASL.

Although several studies have compared the clinical performance of ASL and DSC, few studies have compared their quantitative performance with PET. The comparison studies in gliomas focused on ASL vs. DSC differences in tumor grading (26) or molecular characteristics (27), and the only direct comparison with PET in gliomas used pulsed ASL and performed relative PET measurements only (28). Aside from reporting a similar slight overestimation of CBF with ASL, Ludeman et al.’s results are not directly comparable to ours. Here, we found that the maximum CBF values were, on average, consistent with ^15^O-H_2_O-PET for both ASL and DSC, despite a slight trend of overestimation in ASL. Although the correlation was relatively good, ASL and DSC still cannot fully reproduce PET results. Individual values, however, were rather variable. In the literature, intensity normalization is used to reduce this variability, with the contralateral WM commonly used as a reference region due to its simple delineation. In our study, normalization performed nearly equally well for WM, GM, and contralateral ROI for DSC. But for ASL, normalization of tumor CBF to WM decreased agreement with PET. This is similar to the results in normal tissue, but it partly contradicts previous findings in normalized tumor CBF (4). This could be partly explained by underestimation and noise in WM perfusion due to its longer arterial transit time (29). Also, this study used 2D EPI acquisition with a relatively narrow point-spread function and normalization to deep WM (24). Other studies using 3D acquisitions and normalizing to total WM might have been more contaminated by GM CBF in the reference region because of the greater slice-direction blurring common in 3D ASL (30). Of note, the more commonly used parameter for glioma grade prediction and treatment response evaluation based on DSC is cerebral blood volume (CBV) rather than CBF (31). CBF is related to CBV through the central volume principle, but mean transit times differ across glioma, and there is generally no proportional relation between CBF and DSC-CBV. In any case, CBF is the fundamentally relevant physiological parameter, and DSC-CBV does not offer a major advantage in glioma classification over DSC-CBF (27, 32). Therefore, DSC-CBF was chosen for comparison with ASL-CBF as they both directly assess blood flow, although single-PLD ASL cannot estimate mean transit time and does not offer a fully equivalent model to DSC-CBF.

Given the current small participant group, a preliminary conclusion, consistent with previous ASL vs. DSC comparisons (12, 33), is that ASL and DSC are imperfectly correlated with ^15^O-H_2_O-PET at both regional and voxel-wise levels. Consistency across all three modalities could be demonstrated in only three cases, none of which showed hyperperfusion. Otherwise, hyperperfusion varied regionally, both quantitatively and qualitatively, when assessed visually, with discrepancies across all three techniques. Notably, contrast enhancement was not perfectly associated with hyperperfusion, nor was the absence of enhancement a guarantee for normal perfusion. A weakness of DSC is its reduced sensitivity to smaller microvessels in gradient-echo DSC (34). These are not an issue for ASL or ^15^O-H_2_O-PET, which are insensitive to vessel diameter. This corresponds to our findings in oligodendroglioma, where a “chicken-wire” network of tiny capillaries is typically observed (35), and might also explain the lower DSC signal in grade-2 astrocytoma, which tends to have smaller vessel diameter (36). ASL, on the other hand, is sensitive to late label arrival and tends to overestimate CBF and pick up vascular signals. It should be noted that the current study used single-PLD ASL, which is more prone to macrovascular artifacts, and further studies using multi-PLD ASL acquisition can both reduce these artifacts and provide more information about local variations in label arrival. The most complex case here was a participant with a grade-2 astrocytoma. While the absence of hyperperfusion on DSC might be explained by insensitivity to thinner microvessels, the very high ASL signal had no counterpart in PET. We hypothesize the presence of dense tumor microvasculature with a delayed arrival through collateral vessels. Moderate CBF would then appear similar to GM CBF on PET but as very high CBF on ASL. In practice, this could only be verified using multi-PLD ASL acquisition.

The main limitation is the small study size, owing to the difficulty of performing ^15^O-H_2_O-PET measurements with arterial-blood sampling. The main strength of this study - a heterogeneous population with known differences in vasculature between the included tumor types - is at the same time its main potential limitation. While it increases the clinical relevance of the comparison, a more homogeneous patient cohort would have yielded more homogeneous results. Notably, this population is the first subgroup of a larger study recruiting consecutive patients; thus, patient selection was unbiased. Moreover, oligodendroglioma, astrocytoma, and glioblastoma have previously shown a correlation between malignancy and perfusion and are thus of interest for this comparison (4, 35). It should also be noted that the M0 of tumor tissue may differ from that of blood, which may bias CBF analyses using M0 images; we did not study this effect. Next, the DSC protocol was constructed before the consensus recommendations were reported (15), and the measurements presented here used a longer TR (1900 ms) than recommended (1000–1500 ms) to allow full brain coverage. Still, based on previous simulations, the suboptimal temporal resolution of the data presented here is not expected to have a major effect on our results (15). Slice thickness and resolution differed across all three sequences. While differences in partial-volume effects could lead to regional differences, it is unlikely that this contributed significantly to the general comparability of the studied sequences. Although most ASL research is performed with 3T MRI scanners, many institutions still rely on 1.5T scanners. While ASL offers a better signal-to-noise ratio at 3T than at 1.5T (37), the main issue with ASL comparability with PET in gliomas seems to be the arterial arrival time. Therefore, the results presented here can be generalized to 1.5T as well. Finally, the evaluation of influence on diagnostic findings was outside the scope of the current study.

## Conclusions

In conclusion, while neither ASL nor DSC was fully comparable to ^15^O-H_2_O-PET in all tumors, ASL CBF offers a viable non-invasive alternative to DSC, comparable to ^15^O-H_2_O-PET CBF. However, the possibility of CBF overestimation in ASL images due to macrovascular signal has to be taken into account. Moreover, ASL may provide complementary information to DSC due to its sensitivity to vascular changes, prompting further research to assess its value as a physiological biomarker in the workup of gliomas.

## Data Availability

The data are not publicly available because participants did not consent to public data sharing. Data may be made available upon reasonable request to the corresponding author, subject to applicable ethical and institutional approvals.
